# Electrical Storm With Incessant Ventricular Tachycardia in a COVID-19 Patient: Review of Current Evidence

**DOI:** 10.7759/cureus.15604

**Published:** 2021-06-11

**Authors:** Andrew V Doodnauth, Ridhima Goel, Lu Chen, Vaibhavi Uppin, Zohra R Malik, Krunal H Patel, Samy I. McFarlane

**Affiliations:** 1 Internal Medicine, State University of New York (SUNY) Downstate Medical Center, Brooklyn, USA; 2 Cardiology, State University of New York (SUNY) Downstate Medical Center, Brooklyn, USA; 3 Electrophysiology, State University of New York (SUNY) Downstate Medical Center, Brooklyn, USA; 4 Internal Medicine, St. John's Episcopal Hospital, Far Rockaway, USA; 5 Medicine and Endocrinology, State University of New York (SUNY) Downstate Medical Center, Brooklyn, USA

**Keywords:** covid-19, myocarditis, ventricular arrhythmias, monomorphic ventricular tachycardia, electrical storm

## Abstract

Coronavirus disease 2019 (COVID-19) is associated with various cardiovascular manifestations, including myocarditis, myocardial infarction, and arrhythmias. A prothrombotic state is the primary underlying pathogenic mechanism. While cardiac arrhythmias manifest more commonly amongst critically ill COVID-19 populations, ventricular arrhythmias have been reported only in few cases. This report describes a case of a 95-year-old African American man with COVID-19, who developed sustained monomorphic ventricular tachycardia, which progressed to an electrical storm. The case highlights the importance of high clinical suspicion, early recognition of electrical abnormalities in patients with active COVID-19 infection, and its ability to precipitate fatal ventricular arrhythmia. Also, we provide a literature review on the electrical storm in COVID-19 patients, highlighting the pathophysiologic mechanisms and the management of this deadly arrhythmia.

## Introduction

In addition to acute respiratory complications, coronavirus disease 2019 (COVID-19) has a significant adverse impact on the cardiovascular system, resulting in an increasing prevalence of COVID-19 patients developing severe cardiac complications such as clinically significant arrhythmia. To date, a substantial proportion of reported arrhythmias include atrial and supraventricular tachyarrhythmia. Reports of ventricular arrhythmias have been scarce. We report a case of a COVID-19 patient who developed sustained monomorphic ventricular tachycardia, which progressed to an electrical storm with no known prior medical history of left ventricular dysfunction or coronary artery disease. Additionally, we discuss plausible underlying mechanisms that may have precipitated the inciting event to highlight the importance of ventricular arrhythmia in COVID-19 patients.

## Case presentation

A 95-year-old African American male was found lying in bed with shortness of breath and generalized weakness. Past medical history is significant for advanced dementia, hypertension, multiple prior cerebrovascular accidents (CVA), bed-bound, alert, and oriented at baseline. Emergency medical services were activated and found the patient in a wide complex tachycardia with heart rate (HR) in the 190s and hypotensive with systolic blood pressure (BP) in the 70s.

He received intravenous fluids immediately and underwent direct current cardioversion (DCCV). In the emergency department, his vitals were stable, and he remained in a normal sinus rhythm. He tested positive for COVID-19 according to the nasopharyngeal BioFire® COVID-19 polymerase chain reaction (PCR) test (BioFire Diagnostics, Salt Lake City, Utah). Per his family’s request, he returned home, as he had no oxygen requirements and was otherwise hemodynamically stable.

**Figure 1:**
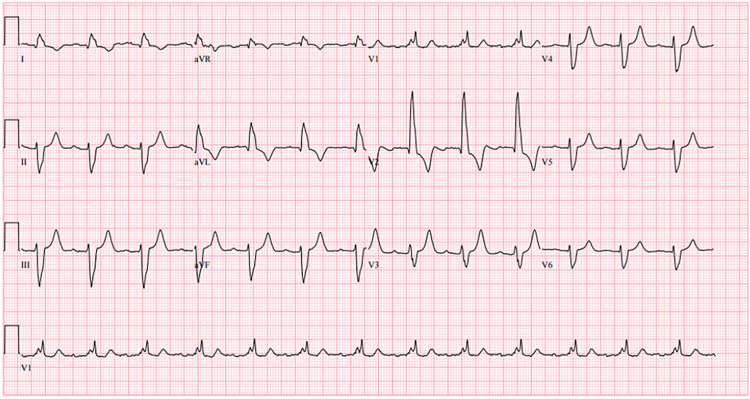
Electrocardiogram from the initial presentation Showing normal sinus rhythm and first-degree atrioventricular-block, bifascicular block, with heart rate 79 beats per minute, and QTc 497 milliseconds

The patient met the criteria to receive treatment for active COVID-19 infection with monoclonal antibodies, bamlanivimab. The following day, after the transfusion, he was found to be tachycardic to the 150s. Nursing staff notified the emergency department and immediately transferred for further management. The patient remained asymptomatic during this time. On arrival at the emergency department, the patient denied any symptoms. He was vitally stable except for tachycardia of 158 beats per minute (bpm). The patient was still alert and oriented, in no acute distress, and his physical exam was otherwise benign, but his increased heart rate was concerning for a potentially fatal ventricular tachyarrhythmia. An electrocardiogram performed in the emergency department on initial presentation the previous day had shown normal sinus rhythm with a first-degree atrioventricular block and bifascicular block (Figure 1).

**Figure 2:**
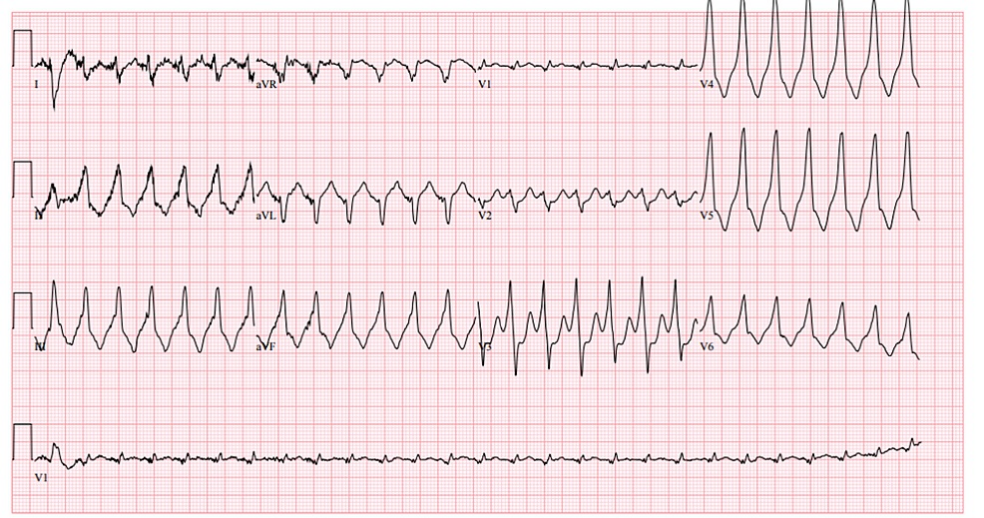
Electrocardiogram on repeat presentation Showing wide complex tachycardia with possible fusion complexes; heart rate 162 beats per minute

In comparison, an electrocardiogram on repeat presentation to the emergency department showed wide complex tachycardia with possible fusion complexes (Figure 2). There was a concern for ventricular tachycardia versus supraventricular tachycardia with aberrancy. Given his presentation, the team decided to treat him for ventricular tachycardia. He was given a loading dose of amiodarone and lidocaine without any resolution of his tachycardia. He then received fentanyl and underwent DCCV at 120 joules, returning to normal sinus rhythm, HR to 70s, and BP 130s/90s.

**Figure 3:**
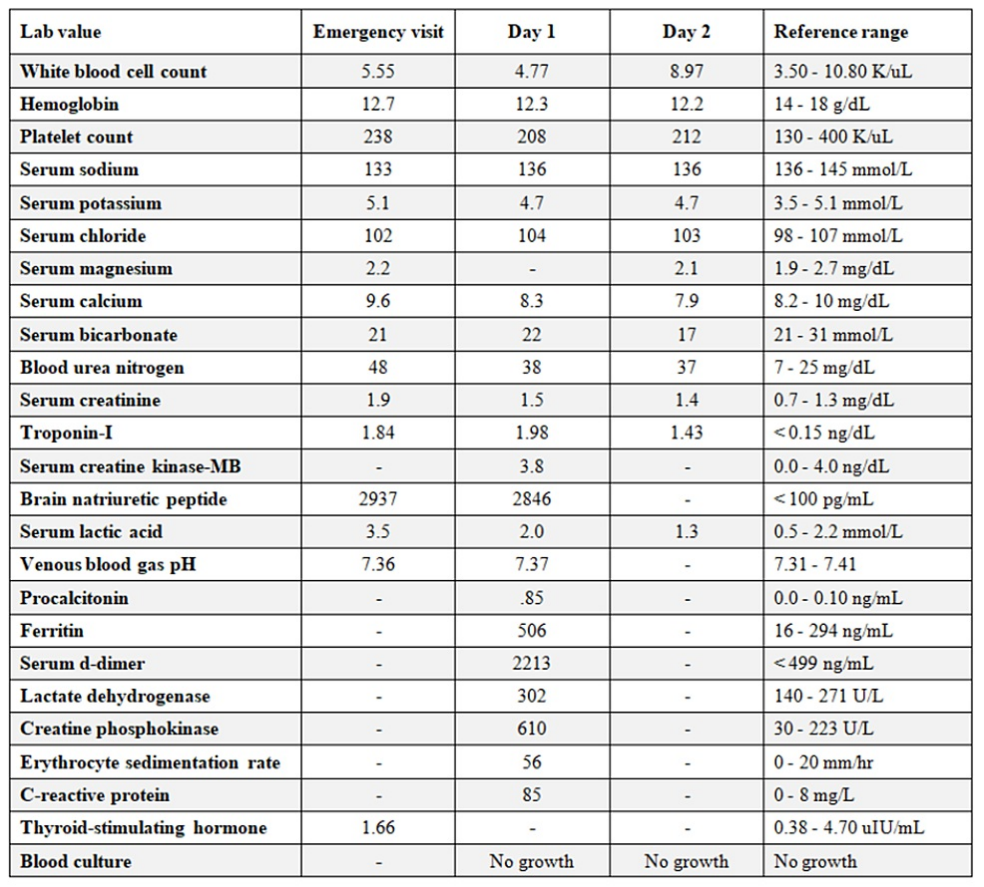
Laboratory results during hospital admission

**Figure 4:**
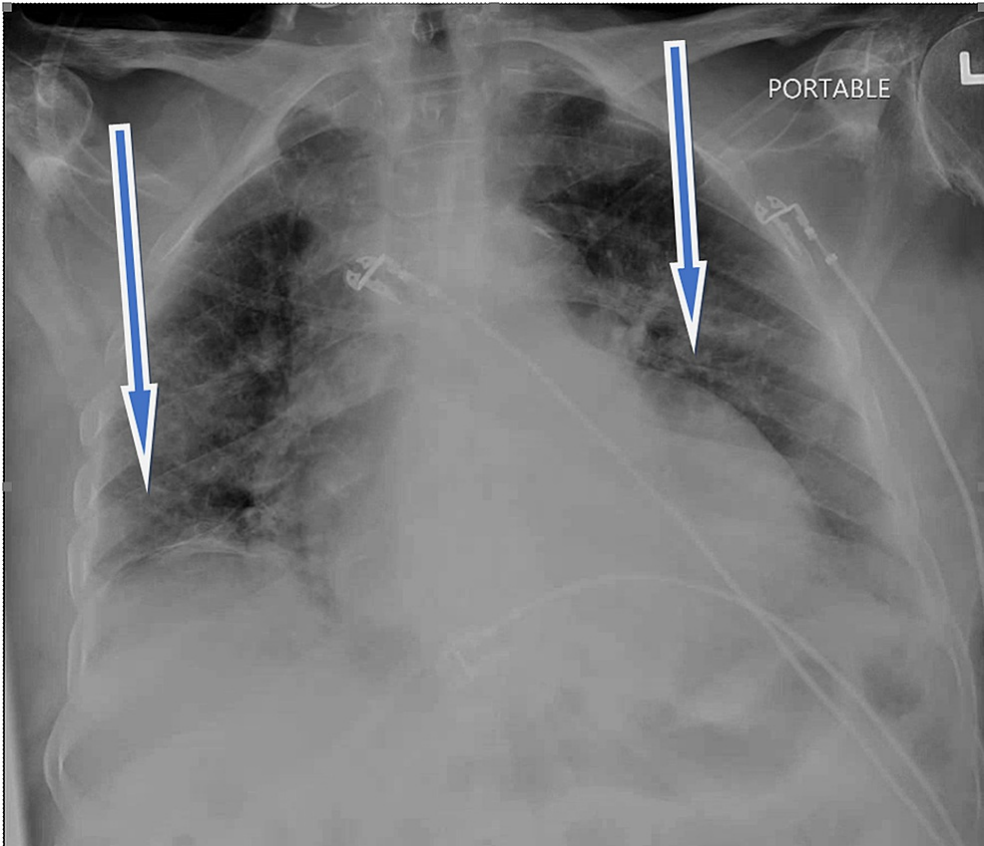
CXR showing bilateral infiltrates (blue arrows) CXR: chest X-ray

Unfortunately, the patient went back into a wide complex tachycardia at a rate of 150-160 bpm spontaneously. His mental status remained intact but became hypotensive, requiring repeat DCCV for a second time to return to normal sinus rhythm. Subsequently, he became hypoxic, requiring placement on a high-flow nasal cannula at 50 liters. Due to an increase in oxygen requirement per hospital protocol, the patient received intravenous dexamethasone and oral remdesivir therapy. He was also loaded with dual antiplatelet agents (aspirin 325 mg and clopidogrel 300 mg) and started on an unfractionated heparin drip due to concern for non-ST elevation myocardial infarction (NSTEMI). Significant initial laboratory values returned a brain natriuretic peptide (BNP) = 2,845 (< 100 pg/mL), troponin-I = 1.98 (<0.15 ng/mL), lactic acid = 2.0 (0.5 - 1.6 mmol/L) (Figure 3). Chest radiograph showed bilateral infiltrates (Figure 4) with pulmonary congestion consistent with atypical pneumonia with superimposed pulmonary edema.

Differential diagnoses included but were not limited to viral myocarditis, ischemic cardiomyopathy, acute decompensated heart failure, type 2 myocardial infarction, pulmonary embolism, and acute hypoxic respiratory failure secondary to COVID-19 pneumonia.

Despite being on an amiodarone drip, the ventricular tachycardia progressed into an electrical storm, for which the patient was started on propranolol and required multiple rounds of DCCV for stabilization, with an eventual return to normal sinus rhythm. Transthoracic echocardiogram (TTE) revealed a mildly dilated left ventricle with markedly reduced systolic function, global hypokinesia, no clinically significant valvular abnormalities, and an estimated left ventricular ejection fraction of 20%.

For the remainder of his hospital admission, the patient remained hemodynamically stable and eventually weaned off high-flow oxygen to the nasal cannula. Before being downgraded to general medicine, the patient transitioned to oral amiodarone therapy.

He eventually maintained adequate oxygen saturation on room air and was safely discharged home in a stable condition. He completed the remainder of his remdesivir treatment as an outpatient.

## Discussion

The American College of Cardiology (ACC) and American Heart Association (AHA) task force defines an electrical ventricular storm as a state of cardiac electrical instability with three or more episodes of ventricular tachycardia occurring within 24 hours [1]. The primary treatment of ventricular tachycardia lies in identifying the precipitating cause of the electrical instability. Common causes include QTc prolonging drugs, electrolyte alterations, myocardial infarction, thyrotoxicosis, hypoxia, increased sympathetic drive, and cardiac channelopathies. There is an emerging correlation with COVID-19 infection leading to an increased predisposition to cardiac arrhythmias in the affected population [2].

Our patient’s original electrocardiogram in the emergency department had been significant for QTc 497 milliseconds. We conducted a thorough reconciliation of his home medications and did not find him on any QT-prolonging agents. A study of COVID-19 patients admitted at the Northwell hospital system in New York City, NY, USA, reported that 6.1% of patients had an initial QTc >500 [3]. Hypokalemia and hypomagnesemia are known to prolong the QT-intervals and precipitate ventricular tachycardia. However, our patient did not have any electrolyte abnormalities and no thyrotoxicosis during his hospitalization.

On further evaluation, there was low clinical suspicion for evidence of myocardial ischemia, as he had no prior history of coronary artery disease and no ischemic changes on his electrocardiogram. The team did not pursue cardiac magnetic resonance imaging because the patient’s TTE demonstrated a global hypokinesis pattern with reduced ejection fraction, perhaps highlighting underlying viral myocarditis. Complete workup for sustained monomorphic ventricular tachycardia includes coronary angiography to assess coronary anatomy. Due to a guarded prognosis and family decision, the patient did not undergo diagnostic cardiac catheterization.

Our patient developed his first episode of ventricular tachycardia before receiving an infusion of severe acute respiratory syndrome coronavirus 2 (SARS-CoV-2) neutralizing antibody LY-CoV555, bamlanivimab, to treat COVID-19; to the best of the authors’ knowledge, no studies so far have reported any arrhythmogenic events in patients receiving this antibody [4]. 

The underlying pathophysiologies of cardiac arrhythmias in COVID-19 patients are multifactorial and not completely understood. It’s believed that the SARS-CoV-2 virus damages the cardiac myocytes by many mechanisms that have arrhythmogenic manifestations [5-6].

Angiotensin-converting enzyme (ACE)-2 receptor-mediated direct cardiac damage

The SARS-CoV-2 virus has an increased predisposition to the ACE-2 receptor, a membrane-bound receptor involved in regulating the renin-angiotensin-aldosterone system (RAAS). Due to its expression on cardiac cells, the virus induces direct myocarditis and disrupts RAAS equilibrium, which can precipitate electrical imbalances leading to arrhythmias [7-8].

Systemic inflammatory response syndrome

COVID-19 increases serum cytokine levels, most profoundly interleukin-6, and causes an imbalance of type 1 and type 2 T-helper cells. A hyperinflammatory state and increased sympathetic activity are both implicated in cardiac arrhythmias [9-10].

Autoimmune cardiac channelopathies

It has also been suggested that the increased immunological responses induce autoimmune antibodies against the cardiac calcium channels that may precipitate electrical abnormalities [11].

Cardiac vascular damage

The SARS-CoV-2 virus propounds a hypercoagulable state and leads to widespread microvascular thrombosis, microangiopathy, and endotheliitis, all of which have been implicated in damage of the cardiac vasculature, resulting in ischemia and leading to ventricular tachycardia [12-13].

Hypoxia-induced myocyte injury

The acute respiratory distress caused by COVID-19 pulmonary disease induces profound hypoxia, exacerbated by reduced adenosine triphosphate (ATP) hydrolysis, energy depletion, and intracellular acidosis, all of which cause direct myocyte damage and contribute to subendocardial ischemia [14].

Pressure/volume changes in the cardiac cycle

The imbalances in the oxygen supply-demand chain, increased vascular thrombi, and pulmonary disease induce right ventricular dilatation, eventually resulting in left and right ventricular diastolic dysfunction [15]. 

All of these mechanisms increase cardiac arrhythmogenicity and predispose patients with an active COVID-19 infection to develop arrhythmias. Just the same, current literary evidence has shown that these patients have a greater proclivity for atrial as compared to ventricular arrhythmias. In a study from Wuhan, China, of 187 patients admitted with COVID-19, the authors reported 11 case reports with ventricular tachycardia/ventricular fibrillation [16]. In a similar study of 700 US patients, the authors reported only 10 patients with non-sustained ventricular tachycardia but no ventricular fibrillation, supraventricular tachycardia, or electrical storm [17]. In a global report of patients hospitalized with COVID-19, Coromilas et al. describe that of the 4,526 patients included in their study, only 67 patients developed ventricular tachycardia [2]. The authors further detail that only 38% of the patients who developed any ventricular arrhythmia were discharged alive. Furthermore, of the total 1,420 patients that died in hospital, 35 of them had ventricular tachycardia or ventricular fibrillation as the recorded rhythm at the time of their death.

Notably, our patient may have had undiagnosed heart failure with reduced ejection fraction, which most likely precipitated his symptoms when superimposed by a systemic inflammatory response. Concomitant cardiac diseases have been associated with worse outcomes in COVID-19 patients and may be supposed to have contributed to our patient’s overall severity of symptoms. Although our patient received antiarrhythmic medication, we believe his ability to maintain normal sinus rhythm and retain electrical stability was due to early aggressive COVID-19 therapy with bamlanivimab, dexamethasone, and remdesivir.

There are still many unknowns regarding COVID-19 infection. Its adverse impact on cardiac electrical circuits, either direct or indirect, cannot be denied. The above patient’s case is among the few reports of electrical storm complicating this vulnerable population of patients with COVID-19. Therefore, this case warrants to be highlighted, as the treating physicians must be aware of this rare manifestation of COVID-19 infection and that approaching them with aggressive management of arrhythmias and COVID-19 can resolve symptoms.

## Conclusions

In about a year, COVID-19 infection has infected millions of subjects and led to widespread mortality worldwide. Additionally, it is associated with severe comorbidities, many of which are still coming to light. While the SARS-CoV-2 virus is considered primarily to cause an infection of the respiratory tract, its impact on the cardiac electrical circuits leading to ventricular arrhythmias is a rare complication that treating physicians must consider. The differential diagnosis for the sudden development of an electrical storm in a previously stable patient remains broad. However, in the setting of active COVID-19 infection, aggressive COVID-19 treatment alone may resolve these symptoms.
